# Inoculation of Goats, Sheep, and Horses with MERS-CoV Does Not Result in Productive Viral Shedding

**DOI:** 10.3390/v8080230

**Published:** 2016-08-19

**Authors:** Danielle R. Adney, Vienna R. Brown, Stephanie M. Porter, Helle Bielefeldt-Ohmann, Airn E. Hartwig, Richard A. Bowen

**Affiliations:** 1 Department of Microbiology, Immunology and Pathology, Colorado State University, Fort Collins, CO 80521, USA; danielle.adney@colostate.edu (D.R.A.); vienna.brown@colostate.edu (V.R.B.); 2 Department of Biomedical Sciences, Colorado State University, Fort Collins, CO 80523, USA; stephanie.porter@colostate.edu (S.M.P.); airn.hartwig@colostate.edu (A.E.H.); 3 School of Veterinary Science, University of Queensland, Gatton, QLD 4343, Australia; h.bielefeldtohmann1@uq.edu.au

**Keywords:** MERS, horse, goat, sheep, reservoir host

## Abstract

The Middle East respiratory syndrome coronavirus (MERS-CoV) was first recognized in 2012 and can cause severe disease in infected humans. Dromedary camels are the reservoir for the virus, although, other than nasal discharge, these animals do not display any overt clinical disease. Data from in vitro experiments suggest that other livestock such as sheep, goats, and horses might also contribute to viral transmission, although field data has not identified any seropositive animals. In order to understand if these animals could be infected, we challenged young goats and horses and adult sheep with MERS-CoV by intranasal inoculation. Minimal or no virus shedding was detected in all of the animals. During the four weeks following inoculation, neutralizing antibodies were detected in the young goats, but not in sheep or horses.

## 1. Introduction

The Middle East respiratory syndrome coronavirus (MERS-CoV) is an emerging pathogen first described from Saudi Arabia in 2012 [1] that can cause severe respiratory disease and death in roughly 36% of infected humans [2]. There is considerable field and experimental evidence that dromedary camels serve as an important reservoir host involved in transmission to humans [3,4,5,6,7,8], but whether other livestock such as goats, sheep, and horses play a role in transmission has only been assessed indirectly. The virus is phylogenetically similar to betacoronaviruses previously detected in bats and there has been speculation that this disease originated through a cross-species transmission from bats to camels or humans [9,10,11].

Serologic testing of sheep, goats, and cattle from Jordan [12] and Saudi Arabia [13] failed to identify animals with neutralizing antibodies to MERS-CoV. Similarly, horses tested in the United Arab Emirates lacked antibodies to MERS-CoV [14]. Direct contact with dromedaries in Saudi Arabia was found to be independently associated with MERS-CoV illness, while contact with goats, sheep, or horses was not associated with human illness [15]. In vitro studies in which replication of MERS-CoV in cultured cells was assessed have yielded mixed results with respect to species susceptibility. Cells from goats, but not sheep or cattle, supported replication of MERS-CoV [16], and primary equine kidney cells supported virus replication, albeit at lower levels than observed with Vero cells [14]. Transfection of the DPP4 receptor from goats, sheep, and horses into non-permissive mouse or hamster cells allowed replication of MERS-CoV [17,18]. Collectively, these in vitro studies suggest the possibility that some livestock are susceptible to infection, but demonstration of infection in live animals is required to better assess their potential as reservoir hosts.

The objective of this study was to determine if goats, sheep, and horses can be infected with MERS-CoV and assess their potential importance in viral transmission. Goats (*n* = 5) were evaluated for viral shedding, organ burden, and seroconversion and transmission to co-housed goats (*n* = 2). Limited viral shedding was observed without demonstration of viral transmission. Due to the lack of transmission, only viral shedding and serology were evaluated in horses (*n* = 4) and sheep (*n* = 3). These animals did not become productively infected or seroconvert, indicating that such livestock are unlikely to serve as reservoirs for MERS-CoV and are unimportant in viral transmission.

## 2. Materials and Methods

### 2.1. Ethical Statement

These studies were approved by the Animal Care and Use Committee of Colorado State University (approval number 13-4384A) and were conducted in an Association for the Assessment and Accreditation of Laboratory Animal Care, International (AAALAC) approved facility.

### 2.2. Animal Study

Two goats, three sheep, and four horses were purchased in Colorado, USA. Both of the goats were bred on site and gave birth to either two (Doe 1) or three kids (Doe 2). All animals were fed a complete pelleted feed supplemented with hay, and were observed at least once daily for nasal discharge, demeanor, food consumption, and clinical status. Sheep, goat kids and horses were each inoculated intranasally with 1.4 × 10^6^ to 1.9 × 10^6^ plaque-forming units (PFU) of a low passage human isolate of MERS-CoV (strain HCoV-EMC/2012) propagated in Vero E6 cells as described previously [11]. The goat kids were maintained at all times in a room with their mothers, who served as in-contact controls to test for virus transmission. Rectal temperature and nasal swabs were taken daily for seven days.

One goat kid from each doe was euthanized 5 days post-inoculation (DPI) and the remaining kids and mother goats were euthanized on day 28 post-inoculation. The horses and sheep were monitored for viral shedding and seroconversion, and were euthanized on day 28 post-inoculation, with the exception of horse 4, which was euthanized on day 17 due to an injury.

### 2.3. Viral Titration

Samples of nasal secretions were collected by inserting and rotating a swab into each nare and were immediately placed in viral transport medium and frozen until plaque assay was performed. Plaques originating from all animals having low titers of virus were confirmed to be MERS-CoV by immunofluorescence using a rabbit polyclonal antiserum against HCoV-EMC-2012 antigen as a primary antibody.

### 2.4. Histology and Immunohistochemistry

Nasal turbinates, trachea, larynx, and lung samples were collected from two kids (goat 1c and 2a) on day 5 post-infection and frozen for virus titration or fixed in 10% neutral-buffered formalin for greater than 7 days prior to being embedded in paraffin. Tissue sections (hematoxylin/eosin and immunohistochemistry) were prepared and evaluated by a veterinary pathologist as previously described [19]. In order to detect MERS-CoV antigen immunohistochemical analysis was performed with a previously described rabbit polyclonal antiserum against HCoV-EMC-2012 antigen [19,20].

### 2.5. Serology

Serum was collected immediately prior to inoculation and weekly thereafter until necropsy. Neutralizing antibodies in sera were assayed using a plaque reduction neutralization test (PRNT) with a 90% neutralization cutoff as described previously [11].

## 3. Results

### 3.1. Goats

**Figure 1:**
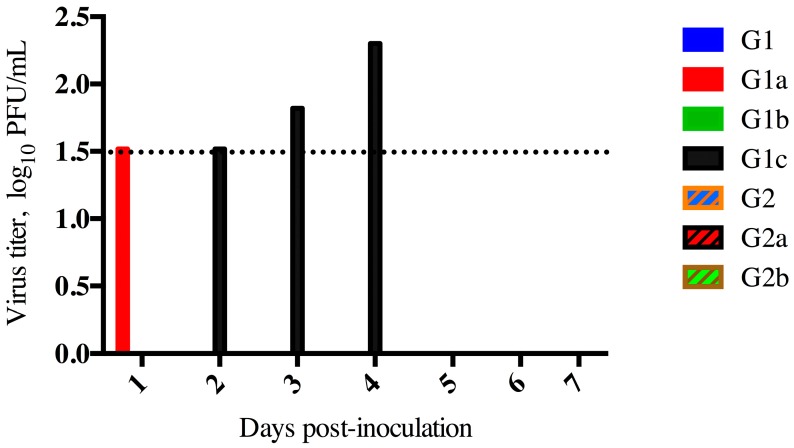
Middle East respiratory syndrome coronavirus (MERS-CoV) shedding in Goats. Virus isolation from nasal swab specimens from experimentally infected goat kids or their co-housed dam. Goat kids 1c and 2a were euthanized day 5 post-infection. The limit of detection for this assay was 1.5 log_10_ PFU/mL, indicated as a dashed line.

Goats were assessed for clinical disease, viral shedding, seroconversion, and viral transmission to their mothers. Fevers were not detected in any of the goats, and no nasal discharge was observed. Low levels of infectious virus were detected in two of the inoculated goat kids from Doe 1 (Figure 1), but not from either of the adult goats that had intimate contact or the kids from Doe 2.

**Figure 2:**
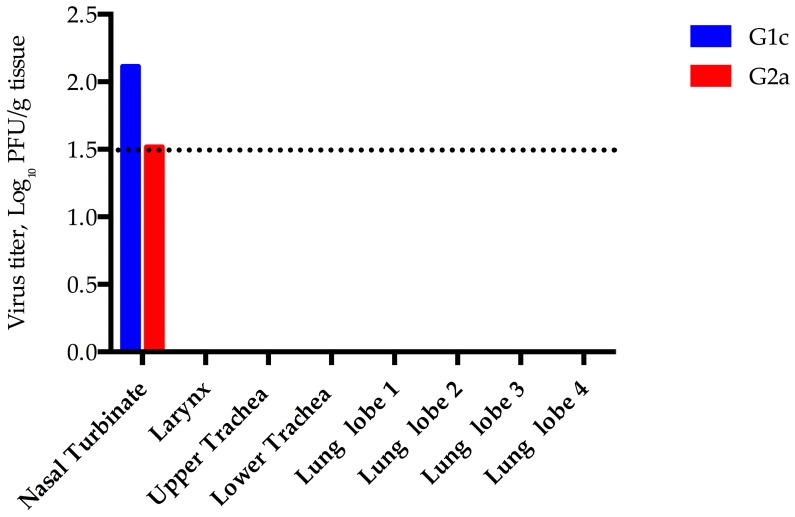
MERS-CoV viral load in goats experimentally infected. Tissues from goat kids 1c and 2a were collected at necropsy 5 days post-infection and viral load was determined by plaque assay of tissue homogenates. The limit of detection for this assay is 1.5 log_10_ PFU/g, indicated by the dashed line.

In order to study acute pathology and determine organ burden, two goats were euthanized on day 5-post infection and nasal turbinates, trachea, and lung were collected. Very small but confirmed quantities of virus were isolated from the turbinates of both goats euthanized 5 days post-infection (DPI) (Figure 2), which may reflect input virus or very low level virus replication. Goat kid 1c was histologically unremarkable, however, the turbinates of goat kid 2a had multifocal areas of loss of goblet cells, epithelial necrosis or squamous metaplasia and attenuation and/or erosion of the epithelium, accompanied by mild to moderate neutrophil and monocyte/macrophage infiltration and occasional minimal hemorrhage. Small amounts of cellular debris, leukocytes and mucus (exudate) were present in the nasal cavity, mainly associated with the aforementioned affected areas. These tissues were negative for viral antigen by immunohistochemistry (IHC) and the histopathologic lesions were very likely the result of trauma from daily swabbing rather than due to virus replication.

The remaining goats were euthanized on day 28 post-infection and the serological status of the experimentally infected kids and their cohoused dams were assessed. Each of three kid goats held past day 5 seroconverted, however, neutralizing antibodies were not detected in either of the dams (Table 1).

### 3.2. Sheep

**Figure 3:**
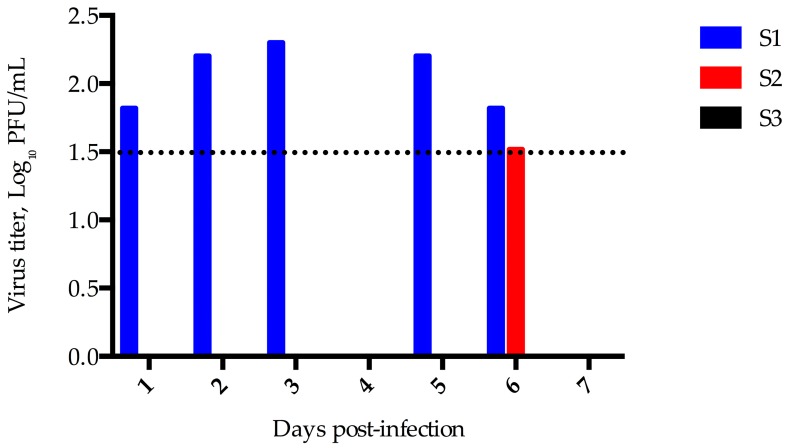
MERS-CoV Shedding in Sheep. Virus isolation was performed by plaque assay from nasal swab specimens obtained from sheep experimentally infected with MERS-CoV. The limit of detection for this assay was 1.5 log_10_ PFU/mL, indicated by the dashed line.

Three sheep were experimentally infected and evaluated for clinical disease, viral shedding, and seroconversion. No nasal discharge or clinical disease was observed and all three sheep maintained consistent food intake and activity levels. A small quantity of virus was detected in nasal swabs from sheep 1 on days 1, 2, 3, 5, and 6 and from sheep 2 on day 6 (Figure 3).

Unlike the goats, acute pathology in sheep was not evaluated in this study. The sheep were euthanized on day 28 post-infection, and serum samples from each week were assessed for the presence of MERS-CoV neutralizing antibodies. Sheep 2 developed a low titer of neutralizing antibody on day 14 (10), but neutralizing antibodies were not detected in either of the other two sheep at any time-point tested (Table 2).

### 3.3. Horses

**Figure 4:**
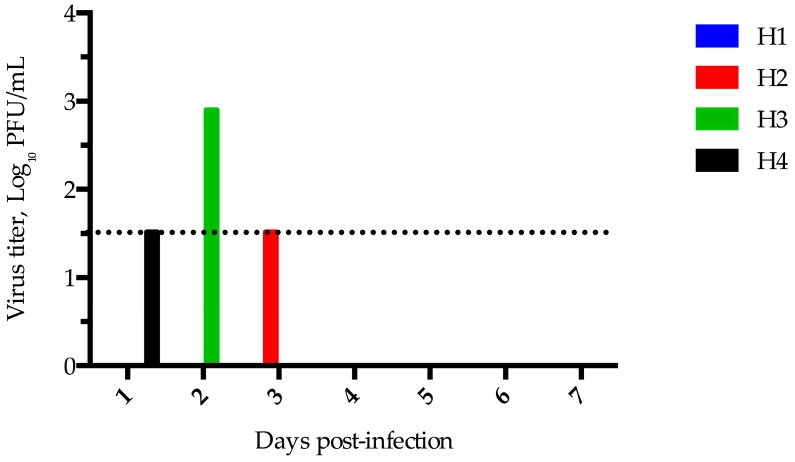
MERS-CoV Shedding in Horses. Virus isolation was performed by plaque assay from nasal swab specimens obtained from horses experimentally infected with MERS-CoV. The limit of detection for this assay is 1.5 log_10_ PFU/mL, indicated by the dashed line.

Despite no detectable rise in rectal temperature or change in appetite and activity, horses 1 and 3 showed mild intermittent nasal discharge prior to inoculation and throughout the 28 days experiment. Low levels of virus were detected in nasal swab samples from three of the four inoculated horses. Virus was detected on day 3 in horse 2, day 2 in horse 3, and day 1 in horse 4 (Figure 4). Virus was not detected in any of the nasal swab specimens tested from horse 1.

Serum was collected weekly until day 28 (with the exception of horse 4, which was euthanized early due to an injury unrelated to the experiment), and evaluated for the presence of MERS-CoV neutralizing antibodies. Unlike the inoculated goat kids, none of the infected horses seroconverted (Table 3).

## 4. Discussion

The objective of this study was to determine if goats, sheep, or horses could be experimentally infected with MERS-CoV. Very limited amounts of infectious virus were detected in nasal swab specimens of some of the experimentally infected animals, but not in uninfected, co-housed goats. It is possible that the infectious virus detected was residual from the input virus, at least on day 1 post-infection. However, a previous study with alpacas re-challenged 80 days after an initial infection was not able to detect infectious virus upon re-challenge indicating that input challenge virus is not detected one day after infection, although the role of secretory antibodies was not addressed in that study [19]. Similarly, another study with alpacas re-challenged on day 28 post-infection was unable to detect viral RNA upon re-challenge [21]. In comparison to experimentally infected dromedaries, significantly less virus was isolated from the livestock in this study. Since the main objective of this study was to determine the role these animals might play in transmission of the virus, we chose to test these samples by plaque assay in order to determine the amount of infectious virus present, rather than by RT-PCR which only reveals the presence of viral RNA regardless of infectivity.

Previous studies of naturally or experimentally infected camels and experimentally infected alpacas showed variable levels of nasal discharge. Studies in camels demonstrated that infected camels have nasal discharge while infected alpacas did not have any observable discharge [8,10,11,19]. The goats and sheep in this study did not have any observable discharge; in contrast horses 1 and 3 had discharge throughout the entire study. All animals were examined and healthy prior to the study, but due to the dust associated with the housing of horses we believe this discharge was unrelated to infection and instead an effect of the environment.

## 5. Conclusions

Current evidence suggests that dromedary camels are the primary reservoir of MERS-CoV. However, elucidating the role that other livestock such as goats, sheep, or horses could play in transmission is important for designing field studies and biosecurity strategies, and in assessing individuals at risk for viral transmission. While in vitro studies suggested that these animals could be naturally or experimental infected, the lack of support from field data coupled with the experimental data presented here suggest that these animals are unlikely to be infected and are not important in viral transmission of MERS-CoV.

## Figures and Tables

**Table 1:** Neutralizing antibody titers in goats experimentally infected or exposed by contact to MERS-CoV. Mother goats are indicated as 1 or 2, and their corresponded kids are indicated as 1a, 1b, 1c (Doe 1), 2a, or 2b (Doe 2). Titers represent dilutions of serum which neutralized ≥90% of input virus. ND = not done.

	1	1a	1b	1c	2	2a	2b
**D0**	<10	<10	<10	<10	<10	<10	<10
**D7**	<10	40	80	ND	<10	ND	20
**D14**	<10	20	40	ND	<10	ND	40
**D21**	<10	20	20	ND	<10	ND	10
**D28**	<10	20	20	ND	<10	ND	<10

**Table 2:** Neutralizing antibody titers in sheep experimentally infected with MERS-CoV. Titers were determined by plaque reduction neutralization test (PRNT) using a 90% cutoff.

	Sheep 1	Sheep 2	Sheep 3
**D0**	<10	<10	<10
**D7**	<10	<10	<10
**D14**	<10	10	<10
**D21**	<10	<10	<10
**D28**	<10	<10	<10

**Table 3:** Neutralizing antibody titers in horses experimentally infected with MERS-CoV. Titers were determined by PRNT using a 90% cutoff.

	Horse 1	Horse 2	Horse 3	Horse 4
**D0**	<10	<10	<10	<10
**D7**	<10	<10	<10	<10
**D14**	<10	<10	<10	<10
**D21**	<10	<10	<10	NA ^1^
**D28**	<10	<10	<10	NA ^1^

^1^ NA: Sample not available.
